# Impact of Pretransplant Donor and Recipient Cytomegalovirus Serostatus on Outcome for Multiple Myeloma Patients Undergoing Reduced Intensity Conditioning Allogeneic Stem Cell Transplantation

**DOI:** 10.4084/MJHID.2013.026

**Published:** 2013-04-10

**Authors:** Jean El-Cheikh, Raynier Devillier, Roberto Crocchiolo, Sabine Fürst, Boris Calmels, Catherine Faucher, Anne Marie Stoppa, Angela Granata, Luca Castagna, Patrick Ladaique, Claude Lemarie, Reda Bouabdallah, Christine Zandotti, Michele Merlin, Pierre Berger, Christian Chabannon, Didier Blaise

**Affiliations:** 1 Unité de Transplantation et de Thérapie Cellulaire (U2T), Institut Paoli-Calmettes, Marseille, France.; 2 Département d’Onco-Hématologie, Institut Paoli-Calmettes, Marseille, France.; 3 Centre de Thérapie Cellulaire, Institut Paoli-Calmettes, Marseille, France.; 4 Département de Microbiologie, Institut Paoli-Calmettes, Marseille, France.; 5 Service de Virologie, Hôpital de la Timone, Marseille, France.

## Abstract

Scope of the study was to investigate the impact of pre-transplant CMV serostatus of the donor and/or recipient on the outcome of patients undergoing allogeneic hematopoietic stem cell transplantation (Allo-SCT) for Multiple Myeloma (MM). To our knowledge no data are available in the literature about this issue.

We retrospectively followed 99 consecutive patients who underwent reduced-intensity conditioning (RIC) Allo-SCT for MM in our cancer center at Marseille between January 2000 and January 2012. Based upon CMV serostatus, patients were classified as low risk (donor [D]−/recipient [R] −) 17 patients (17.1%), intermediate risk (D+/R) 14 patients (14.1%), or high risk – either (D−/R+) 31 patients (31.3%) or (D+/R+), 37 patients (37.3%).

Cumulative incidence of CMV reactivation was 39% with a median time of 61 days (26–318). Three patients (3%) developed CMV disease. Two factors were associated with CMV reactivation: CMV serostatus group (low: 0% vs. intermediate: 29% vs. high: 50%; p=0.001) and the presence of grade II–IV acute GvHD (Hazard Ratio: HR=2.1 [1.1–3.9]). Thirty-six of the 39 patients (92%) with CMV reactivation did not present positive detection of CMV after a 21-day median duration preemptive treatment with ganciclovir. Cumulative incidence of day 100 grade II–IV acute GvHD, 1-year chronic GvHD and day 100 transplantation related mortality (TRM) were 37%, 36% and 9%, respectively. CMV reactivation and serostatus were not associated with increased GvHD and TRM or short survival. Only the presence of acute GvHD as a time dependent variable was significantly associated with increased TRM (p=0.005). Two-year overall and progression free survival were 56% and 34%, respectively.

Donor and recipient CMV serostatus and acute GvHD are independent factors for increased CMV reactivation in high-risk MM patients undergoing RIC Allo-SCT. However, we did not find any influence of CMV reactivation on post transplantation outcome. CMV monitoring and pre-emptive treatment strategy could in part explain these results. Novel prophylactic measures such as immunotherapy and drug prophylaxis need to be considered in this group of patients, warranting further prospective studies.

## Introduction

The introduction of allogeneic hematopoietic stem cell transplantation (Allo-SCT), and novel anti-myeloma agents such as bortezomib, thalidomide and lenalidomide has improved the outcome of patients with multiple myeloma (MM) who relapse or are refractory to standard therapies. 1,2 Allo-SCT at present is considered in the first-line therapy of MM only in young high-risk patients^1^. These advances have transformed myeloma into a chronic condition, with multiple relapses, salvage therapies and chronic immunodeficient states leading to high infection rates. Human cytomegalovirus (CMV) infection remains one of the major complications after Allo-SCT and is associated with considerable morbidity and mortality.^3^,^4^ The incidence of CMV infection increases with the intensity and duration of immunosuppression and occurs in roughly 70% of patients after Allo-SCT, depending on both donor and recipient serostatus.^5^,^6^ Without antiviral intervention, about 50% of patients with culture-proven CMV infection will develop CMV disease. Despite the use of antiviral prophylaxis and pre-emptive antiviral therapy, CMV diseases continue to be reported in 15 to 25% of patients.^7^–^9^ Frequent clinical manifestations of CMV disease are interstitial pneumonitis, gastrointestinal (GI) disease and hepatitis; retinitis, encephalitis and marrow suppression have also been described. The present approach to managing CMV disorders consists of peripheral blood monitoring using either CMV pp65 antigen (antigenemia assay) or polymerase chain reaction (PCR).^10^,^11^

Pre-emptive therapy with either ganciclovir or foscarnet is initiated in patients detected as positive. Factors identified for CMV reactivation are CMV seropositive status of the donor, type of conditioning regimen, use of T-cell depletion, and graft-versus-host disease (GvHD).^12^,^13^ With the introduction of newer conditioning regimens including reduced-intensity conditioning (RIC) Allo-SCT and the increased use of in vivo T-cell depleting agents such as anti-thymocyte globulin (ATG) and monoclonal antibodies such as alemtuzumab (anti-CD52), it has become essential to determine whether a specific high-risk subgroup can be identified.^14^,^15^ These patients could benefit from more intensive or innovative prophylactic strategies as opposed to a general pre-emptive strategy. To our knowledge no data are available in the literature about this issue. We have attempted to study CMV reactivation patterns in 99 consecutive patients with MM who underwent RIC Allo-SCT in our cancer centre at Marseille, where serial CMV monitoring has been done, to ascertain the risks of CMV reactivation.

## Patients and Methods

This is a retrospective analysis of MM patients who underwent RIC Allo-SCT at our centre between January 2000 and January 2012. The data were collected from the transplant database and individual medical records. A total of 99 patients (median age 53 years, range 27–67) underwent RIC Allo-SCT, including 59 males and 40 females. Ninety-six patients (97%) received one or more prior autologous transplantations (Auto-SCT). Twenty-seven patients (27%) were transplanted as the first line treatment strategy whereas 72 patients (73%) had received other treatments. Those 27 patients were considered for Allo-SCT because they had poor prognostic factors like cytogenetic aberrations (Del (13q14), and/or Del 17 and/or t (4; 14)) or they didn’t have at least partial remission (PR) after Auto-SCT. The median time between Auto-SCT and Allo-SCT was 19 months (1–89). The disease status at time of transplantation was Complete Remission (CR) or VGPR in 22%, PR or stable disease (SD) in 67%, and progression or refractory disease (PD) in 11%. Patient characteristics are shown in Table 1.

Seventy-three patients (74%) had matched related donors (MRD) and 26 patients (26%) had unrelated donors (URD). Eighteen patients (18%) had an HLA-allelically matched URD, a so-called 10/10 match, and 8 patients (8%) had 9/10 HLA-matched antigens (four of them had a DQ allele mismatch, two patients had a Cw mismatch and two had A mismatch). The graft source was peripheral blood stem cells (PBSC) in 88 patients (89%), bone marrow in nine patients (9%), and cord blood in two (2%). T-cell depletion with ATG was used in 68 patients (69%) and low-grade total body irradiation (TBI) with 2 Gys was used in 30 recipients (31%) of RIC transplants, respectively. GvHD prophylaxis consisted of cyclosporine A in 56 patients (57%), CSA + mycophenolate mofetyl (MMF) in 41 patients (41%) and MMF alone in two patients (2%). Transplant characteristics are shown in Table 2.

Based upon previous exposure to CMV, patients were classified into low risk (donor and recipient negative; D−/R−), intermediate risk (donor positive with recipient negative; D+/R−), or high risk (recipient positive with donor either negative [D−/R+] or positive [D+/R+]). Seventeen patients (17%) were D−/R−, 14 patients (14%) were D+/R−, 31 patients (31%) were D−/R+ and 37 patients (37%) were D+/R+.

### Supportive care

Our protocol in providing supportive care was the same throughout this time period. Prophylactic treatment against pneumocystis jirovecii and toxoplasmosis consisted of trimethoprim-sulfamethoxazole (10 mg/Kg/day) administered twice weekly. Patients also received daily oral amoxicillin (500 mg × 3/day) as prophylaxis against encapsulated bacteria and prophylaxis against herpes simplex virus including oral valacyclovir (500 mg × 2/day). Patients were monitored for CMV reactivation during the first 12 months after transplant, and pre-emptive therapy with ganciclovir was given if CMV reactivation occurred.

### CMV monitoring and treatment

Serial weekly monitoring for CMV quantification was done using either pp65 antigen (between 2000 and 2009) or a quantitative PCR assay (COBAS R, Roche Diagnostics, Branchburg, New Jersey, USA) (from 2009 to the present). Monitoring was performed weekly initially, starting from transplantation until day 90, and then every 4–8 weeks during the next six months. If there was evidence of reactivation (pp65 >2 cells/200,000 or PCR >1000 copies/mL), treatment was started with ganciclovir (5mg/kg IV twice daily) for 2 weeks, provided two consecutive PCRs done three days apart became negative. If the PCR was still positive after 2 weeks of treatment, a maintenance therapy with ganciclovir (5mg/kg IV once/day) for another 14 days was proposed. CMV disease was diagnosed on demonstration of tissue invasion in biopsy specimens or demonstration of a positive CMV early antigen on bronchoalveolar lavage, along with clinical and radiological features consistent with CMV. *Statistics.* Data are presented as medians, ranges, and 95% confidence intervals (CI). The end points analysed were overall survival (OS), progression free survival (PFS), transplant related mortality (TRM) and acute and chronic GvHD. OS was defined as the time elapsed from Allo-SCT to death, whatever the cause of death. PFS was defined as survival with no evidence of relapse or progression. Kaplan-Meier product-limit estimates were used to assess the probabilities of OS and PFS.^16^ The Prentice estimate and Gray test, allowing the consideration of competing events, were used to calculate the cumulative incidences of GvHD, relapse, CMV reactivation and TRM.^17^,^18^

Cox regression was used to find any association between major pre-transplant variables and OS or PFS;^19^ the occurrence of CMV reactivation, acute GvHD and chronic GvHD were all considered as time-dependent covariates. If two or more variables were associated with p<0.20 to each endpoint of interest, then a multivariate model was constructed. SPSS v13.0 and R 2.12.2 were used for the above cited analyses (http://www.R-project.org).

## Results

### CMV reactivation, disease and treatment

**Figure 1:**
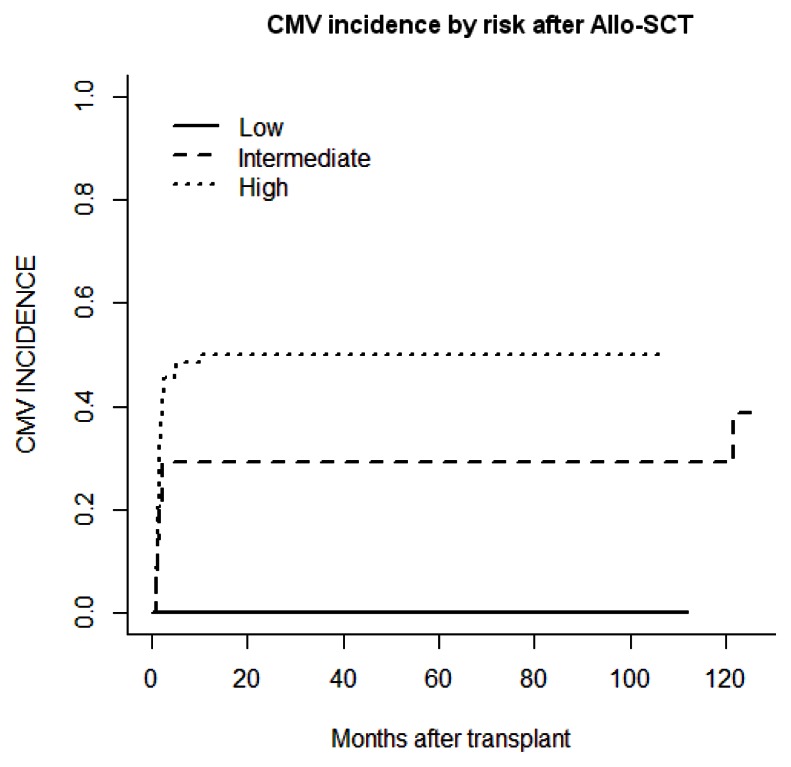
CMV incidence by risk after Allo-SCT

The cumulative incidence of CMV reactivation was 39% (n=39) with a median time of 61 [26–318] days after Allo-SCT. Twenty-three patients (59%) were under corticosteroid treatment for GvHD. There was no difference in the median time (days) of reactivation based upon its method of detection using pp65 (54 [32–162]) or quantitative PCR (65[34–162]). The incidence of CMV reactivation at 1, 3, 6 and 12 months after Allo-SCT was 30%, 35%, 37% and 39%, respectively (Figure 1). Three patients (3%) developed CMV disease (two pneumonitis and one disseminated GI and lung disease) at a median of 38 days (26–49) post Allo-SCT. There was no difference in the median time to reactivation based on donor type (MRD versus URD; 48 versus 46 days), graft source - bone marrow (45 days) versus PBSC (47 days) versus cord blood (46 days) - the year of transplant (before or after 2006), the use of ATG in the conditioning regimen, the age of the donor (more or less than 50 years) or the presence of chronic GvHD.

By univariate analysis, CMV reactivation was significantly influenced by CMV serostatus risk group (high: 50% vs intermediate: 29% vs low: 0%; p=0.001; Table 2 and Figure 1). In the high-risk group characterised by a positive CMV serostatus recipient, the serostatus of the donor (positive vs. negative) did not influence CMV reactivation. The presence of acute GvHD as a time-dependent variable was associated with an increased cumulative incidence of CMV reactivation (p=0.032). Donor type, disease stage, recipient and donor ages, female-to-male graft, graft source (bone marrow versus PBSC versus cord blood), time of transplant (pre- or post-2006), the disease status, the number of lines of previous therapy and time from diagnosis to transplant, the death for other causes, the relapse of the underlying disease, or the presence of chronic GvHD, use of the new anti-myeloma drugs, use of ATG during conditioning and corticosteroid therapy did not significantly influence the incidence of CMV reactivation. Details of the results are shown in Tables 2 and 4.

By multivariate analysis, CMV reactivation was associated with CMV serostatus (HR: 3.8 [1.6–2.9], p=0.002) and the presence of acute GvHD (HR: 2.5 [1.3–4.9], p=0.006).

All 39 patients with CMV reactivation received ganciclovir. Resolution of CMV viremia occurred in 36 patients (92%) after a median of 21 days (range 7–64). Of the three patients who did not have resolution of viremia, all had extensive chronic GvHD. Two of them died of CMV disease while one patient died of disease progression. Recurrent reactivations (after resolution of the first episode with antiviral therapy) were seen in 15 patients, predominantly in patients with chronic GvHD. *Outcome after Allo-SCT.* The median 2-year OS and PFS were 56% and 34%, respectively.

Cumulative incidences of day-100 grade II–IV acute GvHD, 2-year chronic GvHD and 2-year extensive chronic GvHD were 37%, 36% and 29%, respectively. The median TRM at 100 days and one year was 9% and 21%, respectively. All the 10 analyzable pathogenic microorganisms are classified in the table 3 by descending order of frequency.

**Figure 2:**
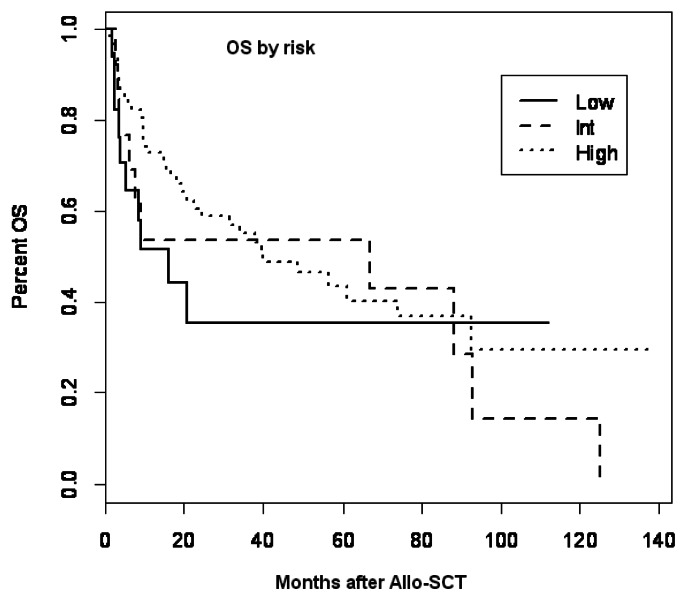
Overall survival (OS) by risk

No impact of pretransplant CMV serostatus on engraftment occurred in any patients. Causes of death were relatively similar between the two groups except for deaths that occurred due to CMV in the group that had reactivation. There was no increase in mortality related to bacterial or other viral infections in patients with CMV reactivation. By univariate analysis, CMV serostatus (low risk versus intermediate risk versus high risk) had no impact on the OS (Figure 2).

Other factors, although evaluated, such as donor type (MRD vs. URD), disease stage, patient and donor ages, female-to-male graft, graft source (bone marrow versus PBSC versus cord blood, time of transplant (before or after 2006), use of the new anti-myeloma drugs, use of ATG during conditioning and corticosteroid therapy did not significantly influence OS, PFS or TRM. CMV reactivation analysed as a time dependent variable did not influence OS (HR=1.4 [0.8–2.4]) (Table 4). Only the presence of acute GvHD as a time dependent variable was significantly associated with increased TRM (p=0.005) (Table 4).

## Discussion

Infections in patients with MM represent a clinical challenge. The list of potential pathogens is long and changes over the disease course.^20^ The aim of this study was to discern the effects of pre-transplant CMV serostatus on the outcome of Allo-SCT in MM patients. To the best of our knowledge, this is the first and largest study reporting on the role of CMV serostatus prior to RIC Allo-SCT in MM patients.

CMV infection remains a major problem following Allo-SCT and different groups have adopted different treatment strategies to reduce the morbidity and mortality related to CMV. One strategy was to serially monitor patients in post Allo-SCT for CMV and start pre-emptive therapy with ganciclovir or foscarnet once there was evidence of reactivation.^6^,^8^,^21^ This practice was established in our institution in 2000, and analysis of this pre-emptive strategy suggests that a third of our patients (39%) develop reactivation. Most importantly, the reactivations occurred in the high-risk groups (D−/R+ or D+/R+), suggesting pre-transplant recipient seropositivity is perhaps the most important risk factor for reactivation.^5^,^5^,^22^ This study showed no impact of pre-transplant CMV serostatus on engraftment in the three risk groups. Previous studies suggested that the CMV serostatus of patients was important not only in terms of reactivation but also in terms of worsening TRM and reducing OS.^5^,^22^ However, in our study, we did not observe worse outcomes in the high-risk group as compared with the other groups. Moreover, we did not find any correlation between CMV reactivation and poor outcome. Interestingly grade II–IV acute GvHD was associated with a higher incidence of CMV infection and there was no increase in mortality related to bacterial or other viral infections in patients with CMV reactivation. In addition to the immunodeficiency related to myeloma and its complications, the type of anti-myeloma therapy used also plays a role in the development of infection, but in this study we did not find an impact of the new anti-myeloma drugs on the outcome of patients after Allo-SCT in any of the three risk groups. These therapeutic strategies impact differently on the immune system, predisposing patients to various opportunistic infections.^20^,^23^ A cumulative suppression of cell-mediated immunity is particular to MM, and results from the combined effect of repetitive use of high-dose corticosteroids, the chronic nature of the disease with multiple relapses requiring salvage therapies (almost always containing dexamethasone), and the addition of bortezomib, a powerful immunosuppressive agent or other immunomodulatory agents like thalidomide and lenalidomide in different treatment phases.^20^,^24^,^25^ In addition, all patients treated with bortezomib-containing regimens should receive prophylaxis for HSV and VZV infection.^26^,^27^ Recently, as reported by our group, the introduction of these novel agents was associated with an improved outcome and we did not observe an increased incidence of infectious disease in patients with high risk MM treated with RIC Allo-SCT.^28^ Initial data on RIC transplants seemed to suggest the incidence of CMV infections with RIC is no different from Myeloablative transplantation (MAT) though the onset of CMV disease seemed to be later.^11^,^15^ More recent data suggest there is a higher incidence of CMV reactivation with RIC transplants, more so with the addition of anti-T-cell antibodies to the conditioning regimen.^29^–^31^ The use of MMF for GvHD prophylaxis was also not found to be associated with an increased risk of CMV reactivation, in contrast with limited published data available from both marrow and solid organ transplants that seem to suggest an increased risk with the use of MMF,^6^,^32^ probably because the patients who had received MMF in our study did not receive ATG in the conditioning regimens. Similarly, the use of a URD and the type of graft source did not seem to impact on CMV reactivation. In a study comparing patients undergoing non-MAT from URD with MRD, the incidence of CMV reactivation was slightly higher with URD, though the difference was not statistically significant.^33^ It is possible that the higher number of RIC Allo-SCT done using MRD (47%) rather than URD (26.7%) may have negated the effect of URD on CMV reactivation. Ljungman et al, in a very large study from the EBMT megafile, reported there was a strong influence of donor CMV serostatus on outcome in URD transplantations.^34^ Patients receiving grafts from CMV-seropositive URD had improved OS and EFS and reduced TRM. However, Walker et al, in a large study involving 753 patients, showed that the graft source (cord blood versus PBSC versus bone marrow) did not independently contribute to CMV reactivation and that patient serostatus was the major determinant of reactivation.^35^ The EBMT study could find no effect of donor CMV serostatus on the risk of acute GvHD.^34^ But analyses of donor CMV serostatus on the risk of chronic GvHD gave different results. Recently, at our institution, we did not observe a higher incidence of infections in MM patients who received transplants from URD compared to patients with MRD.^36^ Furthermore, in this study we observed that grade II–IV acute GvHD was associated with a higher incidence of CMV infection and disease. Unlike the EBMT study, our results indicate that only acute GvHD was a unique factor associated with a higher risk of CMV reactivation. This higher risk was found to be independent of the use of URD or the use of ATG in the conditioning regimens. The incidence of CMV disease was 3% and was limited to the high-risk group alone (D+/R+ and D−/R+).

In conclusion, our findings of comparable outcomes in the three CMV risk groups for MM patients prepared with RIC regimens are important because they suggest that CMV pre-transplant serostatus and reactivation has no influence on OS, PFS and TRM. However, novel prophylactic measures such as immunotherapy and drug prophylaxis need to be considered in this specific group of patients after acute GvHD, warranting further prospective studies.

## Figures and Tables

**Table 1:** Patient and transplantation characteristics

Patient and transplantation characteristics	n = 99 (%)
Patients Age (median) [range]	53 years [27–67]
Male gender	59 (60)
Myeloma-subtype	
IgG	52 (53)
IgA	23 (23)
Light Chain	12 (12)
Bence jones	8 (8)
Other	4 (4)
Cytogenetics at diagnosis	
Normal	14 (14)
Del(13) or Del (17) or t(4;14)	24 (24)
NA	61 (62)
Median number of prior chemotherapies before Allo-SCT [range]	2 [1–5]
1 line	27 (27)
2 lines	46 (47)
3 lines	15 (15)
>3 lines	11 (11)
Median number of prior Auto-SCT [range]	2 [1–4]
1	57 (58)
2	31 (31)
> 2	8 (8)
Status of Myeloma at Allo-SCT	
CR	12 (12)
VGPR	10 (10)
PR/SD	66 (67)
PD	11 (11)
Median interval between Auto- and Allo-SCT months [range]	19 [1–89]
Donor type	
MRD	73 (74)
URD	26 (26)
Donor/recipient sex mismatch	48 (48)
ABO compatibility	
Yes	63 (64)
No	36 (36)
Donor/recipient CMV serostatus	
D−/R− (low risk)	17 (17)
D+/R− (intermediate risk)	14 (14)
D+/R+ (high risk)	31 (31)
D−/R+ (high risk)	37 (37)
Donor Median age years (range)	46 (20–71)
Conditioning regimen	
Flu + Bu + ATG	68 (69)
Flu + TBI	25 (25)
Other RIC	6 (6)
GvHD prophylaxis	
CSA	56 (57)
CSA+MMF	41 (41)
MMF	2 (2)
Stem cell source	
Peripheral Blood	88 (89)
Bone Marrow	9 (9)
Cord blood	2 (2)
Stem cell dose median [range]	
CD34+ × 106/kg	5.41 [0.16–12.8]
CD3+ × 106/kg	299 [5–745]

**Legend:** CR, complete remission; VGPR, very good partial remission; PR, partial remission, PD, progressive disease; Flu, fludarabine; Bu, busulfan; ATG, antithymocyte globulin; CR, complete remission; VGPR, very good partial remission; PR, partial remission, PD, progressive disease; MRD, matched related donor; URD, unrelated donor; GVHD, graft versus host disease; CSA, cyclosporine A; MMF, mycophenolate mofetyl.

**Table 2:** CMV reactivation

	Cumulative incidence of CMV reactivation	p
All patients (n=99)	39%	
CMV serostatus		
Low risk (n=17)	0%	0.001
Intermediate risk (n=14)	29%	
High risk (n=68)	50%	
Time from diagnosis to allo-SCT		
<= 24 months (n=45)	31%	0.117
> 24 months (n=54)	45%	
Disease status at allo-SCT		
CR or VGPR (n=23)	40%	0.960
PR or SD or PD (n=76)	38%	
Previous treatment lines		
1 line (n=27)	30%	0.174
2 or more lines (n=72)	42%	
Donor type*		
MRD (n=72)	38%	0.427
URD (n=24)	46%	
Graft source*		
PBSC (n=88)	38%	0.215
Bone marrow (n=8)	63%	
Transplantation period		
<2006 (n=42)	33%	0.368
>=2006 (n=57)	43%	
Conditioning regimen with ATG		
Yes (n=68)	43%	0.164
No (n=31)	29%	
CMV detection method		
pp65 (n=65)	38%	0.819
PCR (n=34)	39%	
Acute GVHD$	HR=2.1 [1.1–3.9]	0.032
Chronic GVHD$	HR=0.8 [0.1–8.6]	0.837

* Patients with allo-SCT from cord blood (n=2) and haploidentical donor (n=1) were excluded of this analysis.

$ The occurrence of GVHD was analyzed as a time dependent variable

**Table 3:** Classification of pathogenic microorganisms recovered from bloodstream in patients with infection related mortality.

Microorganism	No. (%) Of isolates (n=10)

**Virus**	**5 (5%)**
CMV	2
Epstein bar Virus	1
HHV6+ herpes virus	1
BK virus	1

**Gram-positive cocci**	**1 (1%)**
• Staphylococcus haemolyticus	1

**Gram-negative rods**	**3 (3%)**
• Escherichia coli	2
• Pseudomonas	1

**Yeasts**	**1 (1%)**
**Aspergillus fumigatus**	**1**

**Table 4:** Transplantation outcome

	100-day grade II–IV acute GVHD	P	1-year chronic GVHD	P	100-day TRM	P	2-year PFS	P	2-year OS	P
All patients (n=99)	37%		36%		9%		34%		56%	
CMV serostatus										
Low risk (n=17)	53%		35%		17%		31%		36%	
Intermediate risk (n=14)	36%	0.155	23%	0.613	0%	0.144	23%	0.306	54%	0.456
High risk (n=68)	34%		39%		9%		36%		61%	
Donor type*										
MRD (n=72)	36%	0.528	44%	0.038	10%	0.873	35%	0.423	60%	0.124
URD (n=24)	42%		17%		8%		32%		45%	
Graft source*										
PBSC (n=88)	39%	0.385	39%	0.152	10%	0.133	35%	0.191	56%	0.864
Bone marrow (n=8)	25%		13%		0%		25%		63%	
Transplantation period										
<2006 (n=42)	48%	0.078	50%	0.013	12%	0.544	26%	0.319	60%	0.599
>=2006 (n=57)	30%		25%		7%		42%		53%	
Conditioning regimen with ATG										
Yes (n=68)	35%	0.553	33%	0.481	10%	0.546	35%	0.543	55%	0.834
No (n=31)	38%		42%		6%		31%		56%	
Acute GVHD$					HR=3.6 [1.5-8.7]	0.005	HR=1.4 [0.8-2.2]	0.198	HR=1.6 [0.9-2.7]	0.104
Chronic GVHD$					HR=3.0 [0.9-9.8]	0.061	HR=0.9 [0.5-1.6]	0.799	HR=1.2 [0.6-2.1]	0.598
CMV reactivation$	HR=1.1 [0.6-2.0]	0.852	HR=0.8 [0.4-1.6]	0.511	HR=1.3 [0.5-3.2]	0.570	HR=1.3 [0.8-2.1]	0.351	HR=1.4 [0.8-2.4]	0.260

* Patients with cord blood allo-SCT (n=2) and haploidentical donor (n=1) were excluded of this analysis

$ The occurrence of GVHD and CMV reactivation were analyzed as time dependent variables
